# Eye, Ear and Throat Clinic of Prof. A. W. Calhoun, M.D., in the Atlanta Medical College

**Published:** 1882-02

**Authors:** J. D. Wilson

**Affiliations:** Atlanta, Ga.


ATLANTA

### MEDICAL REGISTER.

VOL. I.] FEBRUARY, 1882. [No. 5

### Original.

### EYE, EAR AND THROAT CLINIC OF PROF. A. W. CALHOUN, M.D., IN ATLANTA MEDICAL COLLEGE.

REPORTED BY J. D. WILSON, ATLANTA, GA.

CASE No. 1.—*Mastoid Disease.*—M. E. W., (white) of Fla., æt. 22, presented himself at the clinic October 13, with the region of the left mastoid process very much inflamed, painful and swollen. He has had otorrhœa for the past twelve years, and the inflammation has gradually extended to the mastoid cells, setting up inflammation of the bone and overlying periosteum. Some constitutional disturbance existed, produced in part by the inflammation and loss of sleep.

*Treatment.*—An incision an inch in length and half an inch in depth was made immediately over the mastoid process, cutting down to the bone, and letting out a large quantity of pus. The wound was packed with lint, and this was re-applied daily after syringing with warm salt water. The otorrhœa was treated simply by syringing the ear with the same fluid and keeping it cleansed.

*Note.*—Saw the patient one month afterwards. The wound over the mastoid process had healed, the swelling and inflammation had all subsided, and the discharge from the ear had entirely ceased. As was anticipated, his hearing was materially diminished.

Case No. 2.—*Congenital Cataract in Both Eyes.*—J. H., (col.) of Ga., æt. 3½ months, was brought for treatment October 13. He was born blind, the result of soft cataract in each eye. The child was healthy otherwise, and had no history of hereditary or other trouble.

*Treatment.*—The pupils having previously been dilated with a 1 grain solution of atropia, the child was chloroformed, the lids separated by means of a speculum and the ball steadied by forceps. A small cataract needle was passed through the sclerotic on the temporal side of the ball, one line in rear of the corneo-sclerotic junction and a little above the median line, to avoid the long ciliary artery. The needle point was pushed in front of the lens to the centre of the pupil, and a crucial incision made in the anterior capsule, to allow the aqueous humor access to the lens substance. This operation was made upon both eyes, and the mother directed to keep the pupils dilated by one drop of the atropia sol., three times daily. The same operation was made upon two subsequent occasions, causing the rapid absorption of each lens. That of the left eye is at present almost entirely absorbed. It may be necessary to repeat the operation upon the right eye. The patient will be able to see, but not distinctly, until cataract glasses have been adjusted. This will be done as soon as the child is old enough to exercise care in their use.

Case No. 3.—*Central Corneal Abscess with Hypopyon. Complete Sloughing of the Cornea.*—S. R. B., of Georgia, æt. 53, was exposed to very cold weather in December, resulting in simple corneitis, which, left to itself, soon formed an abscess. When seen, the abscess was progressing rapidly over the cornea, and about a third of the anterior chamber was filled with pus (hypopyon).

*Treatment.*—The anterior chamber was punctured at the lower corneo-sclerotic junction (paracentesis), and with the exit of the aqueous humor a quantity of pus came out. The patient was directed to use hot fomentations to the eye every few hours, and a few drops of a 2 gr. sol. of atropia, four times daily. The patient had become enfeebled, had slight fever, loss of appetite, etc., and notwithstanding the above vigorous measures, with general tonics, the cornea continued to ulcerate slowly until it had almost sloughed off.

Old men are peculiarly liable to these abscesses and ulcerations of the cornea, especially from injuries. Whenever pus accumulates in the anterior chamber it should be let out by means of a puncture, which process, as a rule, stops the progress of the disease. But, at times, as in this case, nothing arrests its course till the entire cornea is gone ; when it heals over, leaving white cicatricial tissue, and, of course, consequent blindness.

Case No. 4.—*Central Corneal Opacity from Purulent Ophthalmia, Obstructing Vision.*—J. H., of Georgia, æt. 80, several years ago had purulent ophthalmia, and consequent ulceration of the cornea, leaving behind a very thick, white corneal opacity directly in front of the pupil, producing blindness.

*Treatment.*—An artificial pupil (iridectomy) was made at the upper and outer side of the iris, making a clear triangular shaped opening behind the transparent portion of the cornea. The eye was then kept bandaged for a week or ten days. The vision was immediately improved and has continued to do so up to the present time. He is now able to see sufficiently well, with the formerly blind eye, to attend to any ordinary business.

Case No. 5.—*Nasal Catarrh.*—C. A. C., of Alabama, presented himself at the clinic December 1st, with nasal catarrh of two year's standing. The discharge was thick, yellow, occasionally mixed with blood and scabs, and excoriated the nostrils.

*Treatment.*—He was directed to cleanse the nostrils thoroughly with warm salt water twice daily, using both the anterior and posterior nasal douche, and follow it immediatly with the following, used in the same way :

R.—Ammoniæ chloridi, - - - ℥iv

Aquæ, - - - - - - - Oj

M. Et. fac. sol. S.—Tablespoonful to douche.

When the nostrils become accustomed to this, use a chlorate of potash sol. of the same strength ; then after a time stop these and alternate between the two following prescriptions:

R.—Glycerini, - - - - - - ℥ij

Tannici acidi—add as long as it will dissolve.

Sig.—Use as directed.

R.—Cupri sulphatis,

Ferri sulphatis, - - - - aa. ʒj

Aquæ, - - - - - - - ℥ij

M. Et fac. sol. Sig.—Begin (with each of the above) with 5 to 10 drops to each doucheful of warm water, and gradually increase strength as high as patient can tolerate.

After alternating between the last two for a time, he may use the following:

R.—Iodoform. pulv., - - - - - ʒj
Extract. gerani, - - - - - gr. x
Acid. carbolic, - - - - gtt. xv
Vaseline, - - - - - - ℥j

M. Et fac. unguentum. Sig.—Saturate absorbent cotton with it and apply up the nostril at night.

Case No. 6.—*Blepharitis and Conjunctivitis in both Eyes.*—Miss M. H., æt. 23, of Georgia, presented herself with the above named troubles December 17.

*Treatment.*—

R.—Hydrarg. oxid. flav., - - - - gr. j
Vaseline, - - - - - - ʒj

M. Et fac. unguentum.

Sig.—Rub a piece on each lower lid nightly.

Also use the following:

R.—Zinci sulphatis.

Aluminis.

Morphiæ, aa, - - - - - - gr. j

Aquæ, - - - - - - - - ℥j

M. Et fac. sol. Sig.—Drop a few drops in eye three times daily.

*Note.*—This treatment was continued for a few weeks and the eyes are now practically well.

Case No. 7.—*Mature Senile Cataract in the Right Eye and Immature in the Left.*—S. W., of Georgia, æt. 63, has been gradually growing blind for more than a year from cataract. In the right eye the lens had become perfectly hardened and opaque (mature cataract)—the patient being able merely to perceive light. The left eye is gradually progressing in the same direction. Inasmuch as the left eye was growing rapidly blind, and the right was already blind, it was decided to remove the cataract from the right eye at once. After dilating the pupil and getting the patient otherwise ready by some preparatory treatment, Graefe's linear extraction was made and the cataractous lens removed with success. He was at once able to count fingers and made a rapid recovery, being confined to bed and room from ten days to two weeks. His vision has not yet been tested, but with proper cataract glasses it ought to be almost normal. Nothing will be done to the left eye until the cataract becomes mature.

Case No. 8.—*Corneal Staphyloma.*—G. A., æt. 12, of Alabama, was brought to the clinic for treatment October 13. As the result of sore eyes last January, he had an ulcer on the left cornea which terminated in a staphyloma of its lower half. When presented it was growing very fast, the vision in that eye being entirely destroyed.

*Treatment.*—Critchett's operation was made, which consists in passing five curved threaded needles through the ball from above downwards, behind the ciliary processes, and the removal of all in front of needles by knife or scissors. The needles are then drawn through and the opening closed by tying the stitches thus made.

He was then directed to use cold water applications occasionally to prevent excessive inflammation. The operation was endured without an anæsthetic.

Case No. 9.—*Parenchymatous Keratitis in Both Eyes.*—J. T., æt. 12, of Georgia, presented himself for treatment November 10th. He had suffered from the above disease for some time, both corneæ being very hazy, and vision consequently much interfered with. The following treatment was instituted:

R.—Atropiæ sulphatis, - - - gr. ij
Aquæ dest., - - - - - ℥j

M. Et fac sol. Sig.—Drop a few drops in eye three times daily. Also use the following:

R.—Potass. iodidi., - - - - - ʒi

Hydrarg. bichlorid., - - - gr. i

Aquæ dest., - - - - - ℥vi

M. Et fac. sol. Sig.—One teaspoonful three times a day, gradually increasing up to two.

*Note.*—These cases are apt to be a little slow, but with patience and the above treatment, aided by tonics, a cure is almost certainly effected. In the present instance the hazy appearance is gradually clearing up, and in a month or so will probably disappear entirely.

CASE No. 10.—*Pterygium.*—W. K. B., of Ga., æt. 22, came with a pterygium on the nasal side of the left eye.

*Treatment.*—The growth was shaved off from its corneal attachment, and dissected up toward the inner canthus. A diamond-shaped piece was then cut out and the edges brought together by two stitches, a part of the conjunctival surface being left to heal by granulations. The result was a success.

CASE No. 11.—*Ectropion of Both Lower Lids. Operation with Complete Recovery.*—J. Y., of Ga., æt. 55, has had chronic inflammation of the conjunctiva and some long standing skin affection of the face, resulting in contraction of the skin and relaxation of the conjunctiva, causing both lower lids to turn out extensively.

*Treatment.*—The triangular flap operation was made upon the right lower lid, the incision beginning at each canthus and meeting at a point about an inch below the centre of the edge of the lid. This flap was dissected up and the lid brought in proper position, when the entire wound was closed up by stitches. It healed by first intention, the lid remaining as in the natural eye. On the left eye the ectropion was not so great, and the thread operation was made as follows: A needle was placed upon each end of a thread and the two needles passed from the conjunctival surface of the lid, just below its margin—one being near the outer, the other near the inner canthus—and brought out on the skin surface about half an inch below the margin of the lid. The two ends were then tied tolerably tightly over a small stick, which caused the lid to return at once to its natural position. The object of this operation is to excite a sufficient amount of inflammation in the lid substance to contract the conjunctiva and hold it permanently in its place. After three or four days the thread was removed, showing the operation to be a complete success.

